# Nontraumatic Fracture of the Femoral Condylar Prosthesis in a Total Knee Arthroplasty Leading to Mechanical Failure

**DOI:** 10.1155/2014/896348

**Published:** 2014-01-23

**Authors:** Girish N. Swamy, Conal Quah, Elmunzar Bagouri, Nitin P. Badhe

**Affiliations:** Trauma and Orthopaedics, Queens Medical Centre, Nottingham University Hospitals NHS Trust, Nottingham, UK

## Abstract

This paper reports a case of fatigue fracture of the femoral component in a cruciate-retaining cemented total knee arthroplasty (TKA). A 64-year-old man had undergone a primary TKA for osteoarthritis 10 years previously at another institution using the PFC-Sigma prosthesis. The patient recovered fully and was back to his regular activities. He presented with a history of sudden onset pain and locking of the left knee since the preceding three months. There was no history of trauma, and the patient was mobilizing with difficulty using crutches. Radiographs revealed fracture of the posterior condyle of the femoral prosthesis. Revision surgery was performed as an elective procedure revealing the broken prosthesis. The TC3RP-PFC revision prosthesis was used with a medial parapatellar approach. The patient recovered fully without any squeal. Mechanical failure of the knee arthroplasty prosthesis is rare, and nontraumatic fracture of the femoral metallic component has not been reported before.

## 1. Summary and Background

Mechanical failure of prosthesis is a rare but recognised complication in arthroplasty surgery. There are reports of acute breakage of the tibial posts [1–7], tibial polyethylene inserts [8–10] in posterior cruciate substituting total knee arthroplasty, and fracture of metal tibial trays after kinematic total knee replacement [11–13].

We describe a case of nontraumatic fracture of the posterior condyle of the femoral component in a total knee arthroplasty, which to our knowledge is the first report of its kind.

## 2. Case Report 

**Figure 1:**
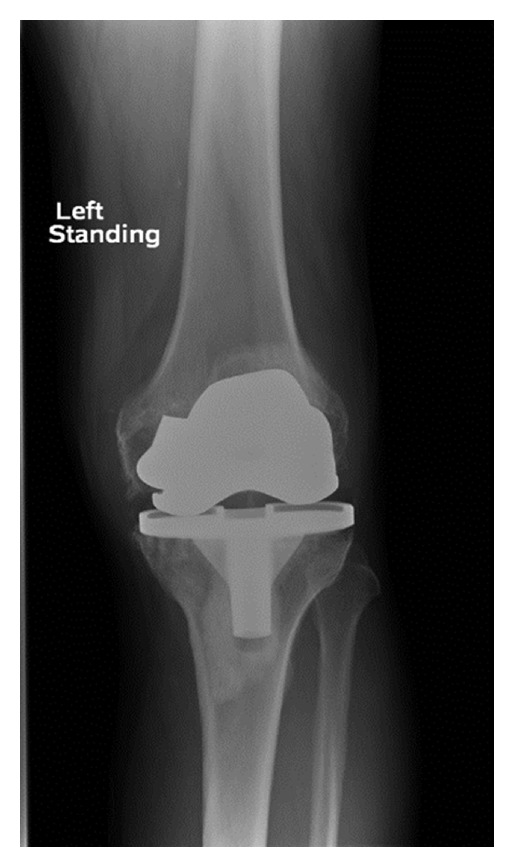
An anteroposterior radiograph of the knee showing evidence of the fracture of the medial femoral condylar prosthesis.

**Figure 2:**
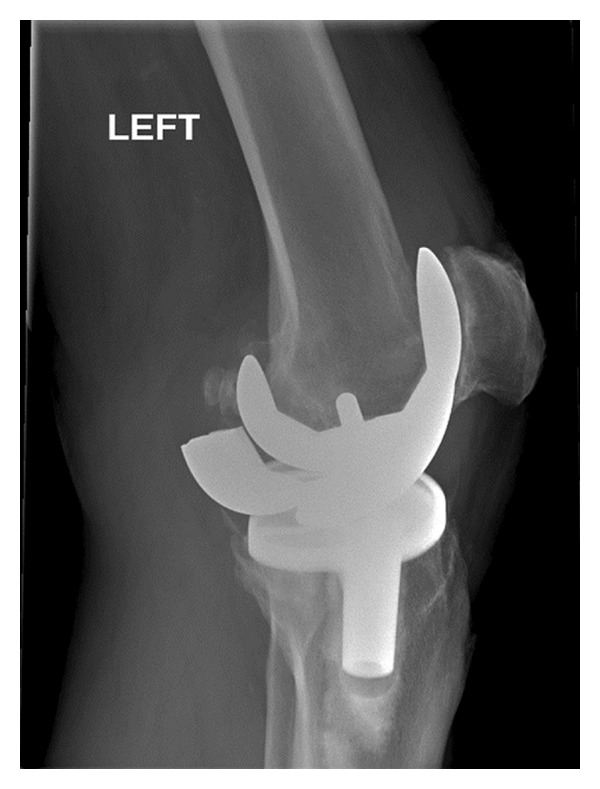
A lateral radiograph of the knee showing the broken prosthesis.

**Figure 3:**
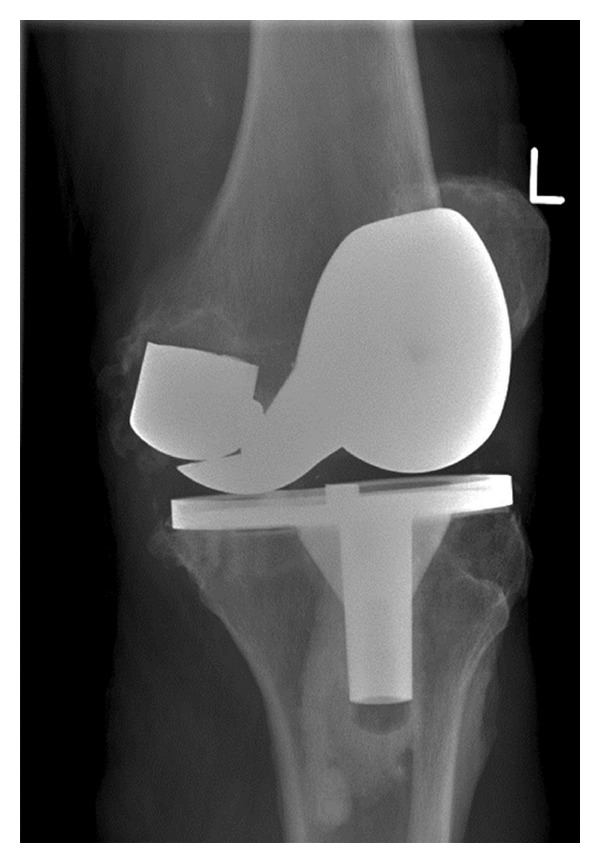
An oblique radiograph of the knee demonstrating the broken prosthesis.

In December 1999, a 64-year-old man underwent a cemented left total knee arthroplasty using a sigma PFC posterior cruciate retaining total knee prosthesis (DePuy Orthopaedics, Warsaw, IN, USA) for degenerative changes in relation to osteoarthritis using a medial parapatellar approach in a different centre. The patient recovered uneventfully and was doing well. In July 2009, the patient presented to us with a history of sudden onset pain and locking of the left knee since the preceding three months. There was no history of trauma and since was mobilising with difficulty using crutches. The patient weighed 89 kgs and had no other medical comorbidities. Clinical examination revealed a severe antalgic gait and a significant amount of rocking with metal on metal noise in 20 degrees flexion. Passive movements ranged from 0 to 90 degrees. At that moment, the knee was quiescent. AP and lateral radiographs (Figures 1, 2, and 3) of the left knee revealed a fracture of the posterior condyle of the femoral prosthesis with rotation and evidence of further loosening. Given the patients symptoms and the radiographic evidence, a decision to perform a revision of the total knee arthroplasty was made after a fully informed written consent.

**Figure 4:**
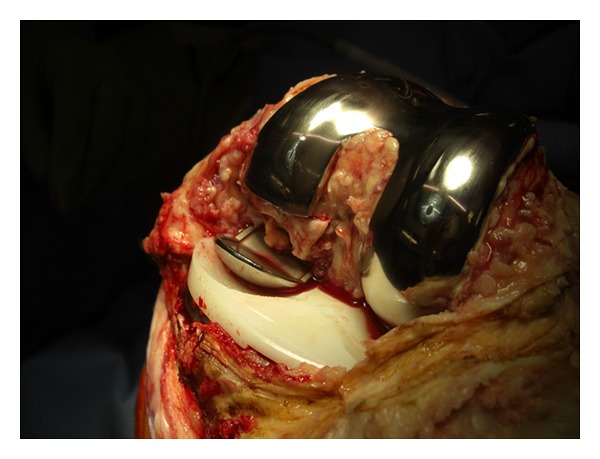
Intraoperative evidence of the fracture through the medial femoral condylar implant.

**Figure 5:**
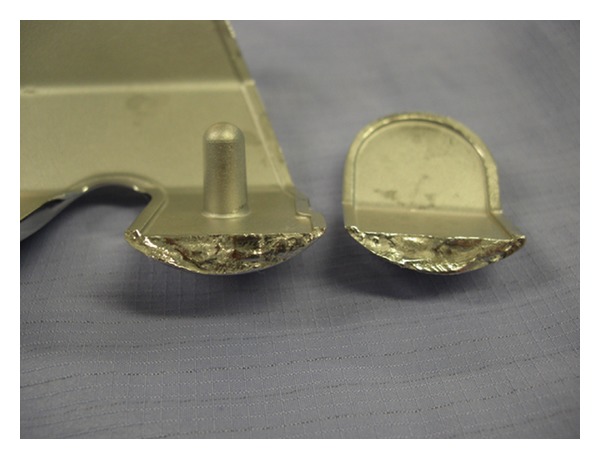
Broken femoral component was cleaned and sent for metallurgical analysis.

Revision surgery was performed as an elective procedure in August 2009 revealing the broken prosthesis (Figures 4 and 5). A TC3 RP PFC revision prosthesis was used with a medial parapatellar approach. A size 4 tibial prosthesis with a 53 mm sleeve and 12∗75 mm stem and a size 4 femur with 31 mm sleeve and 14∗75 mm stem along with femoral metallic augments and a 12.5 mm RP insert were used. Intraoperative range of motion was from 0 to 120 degrees and the procedure was uneventful. The patient recovered well and was mobilised with touch weight bearing for 6 weeks. Checking the X-ray revealed a satisfactory prosthesis and the patient was discharged on the 8th postoperative day. Follow-up at 6-week and 6-month period revealed a satisfactory progression with no instability and the patient was pain free.

A metallurgical analysis of the failed prosthesis revealed no weakness in its strength.

## 3. Discussion

Very few reports exist of fracture of the metallic components after a total knee arthroplasty. Abernethy and coworkers [11] reported on 16 revisions due to the fracture of the metal tibial tray after kinematic total knee replacement. They found a strong association with failure to adequately correct a preoperative varus deformity and the use of bone graft to correct such a deformity with fracture of the metal base plate within four years of implantation. This was probably due to the displacement of the mechanical axis to the medial side of the knee. They also found that the fracture of the metal base plate was the most common cause of aseptic failure of the kinematic condylar knee replacement. Scott et al. [12] have suggested that heavy, active males are at the most risk of base-plate fractures.

Our patient had a near-normal anatomic static alignment of the total knee prosthesis and was also not overweight and not obese. He was though very active and still in a full time job. Mariconda et al. [8] reported on acute breakage of the polyethylene tibial insert in a genesis total knee replacement with flexion of the total knee replacement leading to the development of a downward force on the polyethylene insert and shear force possibly causing the fracture.

Several reports [1–6] exist of the failure of the tibial post in the posteriorstabilised TKA (PS-TKA) using a cam and postmechanism. Although this is a rare case of a fracture of the metallic femoral condylar component in a total knee replacement, the complication is a reminder to us of the possibility and further assessment.
